# microRNA Expression in Women With and Without Polycystic Ovarian Syndrome Matched for Body Mass Index

**DOI:** 10.3389/fendo.2020.00206

**Published:** 2020-04-28

**Authors:** Alexandra E. Butler, Vimal Ramachandran, Thozhukat Sathyapalan, Rhiannon David, Nigel J. Gooderham, Manasi Benurwar, Soha R. Dargham, Shahina Hayat, S. Hani Najafi-Shoushtari, Stephen L. Atkin

**Affiliations:** ^1^Diabetes Research Center (DRC), Qatar Biomedical Research Institute (QBRI), Hamad Bin Khalifa University (HBKU), Qatar Foundation (QF), Doha, Qatar; ^2^Division of Research, Weill Cornell Medical College-Qatar, Qatar Foundation, Education City, Doha, Qatar; ^3^Academic Diabetes, Endocrinology and Metabolism, Hull York Medical School, University of Hull, Hull, United Kingdom; ^4^Department of Surgery & Cancer, Faculty of Medicine, Imperial College, London, United Kingdom; ^5^Department of Cell and Developmental Biology, Weill Cornell Medicine, New York, NY, United States; ^6^Royal College of Surgeons Ireland, Busaiteen, Bahrain

**Keywords:** microRNA, polycystic ovarian syndrome, follicular phase, menstrual cycle, insulin resistance, androgens

## Abstract

**Background:** Despite several authors who have hypothesized that alterations of small noncoding RNAs (miR) are implicated in the etiopathogenesis of polycystic ovarian syndrome (PCOS), contrasting findings have been reported so far. Discrepancies in body mass index (BMI) levels may account for these differences; therefore, the aim of the present study was to determine whether miR differed in serum samples collected from age- and BMI-matched control and PCOS women.

**Methods:** In a cross-sectional study, miR were measured using quantitative polymerase chain reaction in 29 women with anovulatory PCOS women and 29 control women who were in the follicular phase of their menstrual cycle, from the local biobank.

**Results:** One hundred seventy-six miR were detected, of which 15 miR passed the false discovery rate (FDR; *p* < 0.05) that differed between PCOS and control women. There was no association of the top 9 miR (*p* < 0.02) (miR-486-5p, miR-24-3p, miR-19b-3p, miR-22-3p, miR-19a-3p, miR-339-5p, miR-185-5p, miR-101-3p, miR-let-7i-5p) with BMI, androgen levels, insulin resistance, or antimullerian hormone (AMH) in either PCOS or normal women. Ingenuity pathway assessment showed the pathways were interrelated for abnormalities of the reproductive system.

**Conclusion:** When the confounding influence of weight was accounted for, miR levels differed between anovulatory PCOS women and control women in the follicular phase of the menstrual cycle. Interestingly, the differing miR were associated with the pathways of reproductive abnormalities but did not associate with AMH or metabolic parameters.

## Introduction

Polycystic ovarian syndrome (PCOS) is the most common underlying cause of anovulatory infertility in women of reproductive age (1) and is associated with metabolic features that include clinical and biochemical hyperandrogenism, insulin resistance, obesity, type 2 diabetes (T2DM), and hypercholesterolemia (2–5). Epigenetic abnormalities, genetic mutations, and alterations of noncoding RNAs are involved in its etiology (6–9).

microRNAs (miRNAs) are noncoding RNAs of ~22 nucleotides in length that posttranscriptionally regulate gene expression. Their binding to target messenger RNA (mRNA) causes mRNA cleavage, translational repression, and mRNA decay (10–13). Only ~600 of the 2,600 mature human miRNAs described have been confidentially validated as microRNA in the miRBase miRNA registry (14, 15). Due to their multitudinous base pairing, allowing each miRNA sequence to attenuate multiple target mRNA expressions, they affect many different biological/cellular pathways. Their involvement in almost every biological process results in miRNAs being implicated in a number of disease states, including obesity and T2DM, and their associated comorbidities (16, 17). MiRNA have been reported to regulate many biological processes linked with obesity, including lipid metabolism, adipogenesis, insulin secretion, and glucose uptake (18–21).

miRNA expression is highly complex, and therefore, it is not surprising that a number of published studies in PCOS subjects appear discordant (22). MiRNA-21, miRNA-27b, miRNA-103, and miRNA-155 have been reported to be differentially expressed in both PCOS and obesity (23). Serum miRNA-21 (24) and miRNA-6767-5p (25) are elevated, while miRNA-320 is lowered (26), in PCOS and have been proposed as biomarkers for diagnosis of PCOS. In the ovary, miR-92a and miR92b have been described to be differentially expressed in PCOS (27), while in the ovarian cortex of PCOS subjects, miR-93 expression was highest (28). MiR-93 has also been implicated in downregulating the expression of GLUT4, the insulin-sensitive glucose transporter present in adipose tissue (29); miRNA-223 also plays an ill-defined role in the insulin resistance of PCOS. In adipose tissue, miRNA-93 has been reported to be overexpressed in insulin-resistant PCOS women (although, of note, the overexpression was discordant with expression of MCM7, the host gene) (30). A study in Chinese women with and without PCOS reported five upregulated circulating miRNAs (let-7i-3pm, miR-5706, miR-4463, miR-3665, miR-638) and four downregulated miRNAs (miR-124-3p, miR-128, miR-29a-3p, let-7c) in PCOS patients.

In the present study, we utilized serum samples collected by the PCOS Biobank in the follicular phase of the menstrual cycle in control women and compared them to serum samples from anovulatory PCOS subjects. Our objective was to determine whether circulating miRNAs differed between women with and without PCOS when weight and age were matched. Our hypothesis was that there would be no difference in circulating levels of miRNAs between women with and without PCOS when weight and age were matched.

## Materials and Methods

### Study Design

In a cross-sectional study, 29 medication-naive PCOS patients (aged between 18 and 45 years) with biochemical hyperandrogenemia were analyzed from the local PCOS biobank (ISRCTN70196169) having been acquired from patients presenting sequentially to the Endocrinology Department between May 2012 and June 2013. Twenty-nine normal control women (aged between 20 and 44 years) were recruited by advert and were selected to be age and body mass index (BMI) matched to the PCOS subjects, who were all anovulatory for the preceding 6 weeks as criteria for inclusion into this study with a progesterone at the point of study of <5 nmol/L. Demographic data for the PCOS and control women is shown in Table 1. For the purposes of this study, PCOS diagnosis required that all three diagnostic criteria of the Rotterdam consensus be met. Therefore, all PCOS patients had clinical and biochemical evidence of hyperandrogenemia (Ferriman–Gallwey score, >8; free androgen index, >4, respectively), abnormal menses (oligomenorrhea or amenorrhea), and polycystic ovaries documented on transvaginal ultrasound (31). Liver ultrasound was performed in order to exclude nonalcoholic fatty liver disease (NAFLD). None of the study participants had any other illness, nor had they been taking any medication for the preceding 9 months. To exclude diabetes, a 75 g oral glucose tolerance test (OGTT) was administered. Other endocrine disorders (nonclassical 21-hydroxylase deficiency, hyperprolactinaemia, Cushing's disease, and androgen-secreting tumors, for example) were excluded. All patients gave their written informed consent. Approval to perform this study was granted by the Newcastle & North Tyneside Ethics committee. The conduct of the trial was in accordance with International Conference on Harmonization Good Clinical Practice (ICH GCP) and the Declaration of Helsinki.

**Table 1:** Demographics, biochemical, and clinical markers (mean ± SD) for the polycystic ovarian syndrome (PCOS) and control group from biobank patients.

	**Normal patients** ***N*** **=** **29**		**PCOS patients** ***N*** **=** **29**		***p* value**
	**Mean**	**SD**	**Mean**	**SD**	
Age (years)	32.4±	7.8	31.6	8.8	0.735
Median (IQR)	33.5 (29.0–36.3)		30.0 (27.5–34.0)		
BMI	27.2±	6.0	28.8	5.6	0.324
Median (IQR)	25.6 (22.8–28.1)		26.0 (23.1–28.8)		
Glucose (mmo/L)	4.6	0.5	4.8	0.6	0.202
Insulin (mIU/ml)	7.3	4.9	12.0	6.1	0.012
HOMA-IR	1.5	0.9	2.6	1.7	0.006
Testosterone (nmol/L)	1.0	0.4	2.5	1.6	0.007
AMH	23.8	3.2	54.9	4.2	0.001
SHBG (mmol/L)	76.4	76.1	46.6	51.0	0.167
FAI	2.0	1.0	12.4	18.0	0.013

*BMI, body mass index; FAI, free androgen index; SHBG, sex hormone binding globulin; HOMA-IR, homeostatic model assessment-insulin resistance*.

### Sample Collection

Having established that the control women were in the follicular phase of their menstrual cycle, and following an overnight fast, blood samples were drawn, and serum was stored frozen at −80°C, pending analysis. Isotope dilution liquid chromatography–tandem mass spectrometry (Waters Corporation, Manchester, UK) was used to measure serum testosterone levels. An immunometric assay with fluorescence detection (performed on a DPC Immulite 2000 analyzer following the manufacturer-recommended protocol) was used to measure sex hormone binding globulin (SHBG). The free androgen index (FAI) was calculated using the following formula: total testosterone × 100/SHBG. A competitive chemiluminescent immunoassay using a DPC Immulite 2000 analyzer (Euro/DPC, Llanberis, UK) was used to determine serum insulin concentration. For the insulin assay, sensitivity was 2 μU/ml, and the coefficient of variation (CV) was 6%; the manufacturer claimed no cross-reactivity with proinsulin. A Synchron LX 20 analyzer (Beckman-Coulter) was used to measure plasma glucose concentration, following the manufacturer-recommended protocol; during the study period, the assay had a CV of 1.2% at the mean glucose value of 5.3 mmol/L. Insulin resistance was calculated using the accepted homeostatic model assessment (HOMA) method [HOMA-IR = (insulin × glucose)/22.5]; HOMA-β was used to calculate pancreatic beta cell sensitivity [HOMA-β = (20 × insulin)/glucose – 3.5].

### microRNA Isolation

MiRNA was isolated from 200 μl plasma using the miRCURY™ RNA Isolation Kit (Exiqon A/S, Denmark); manufacturer-recommended protocol was followed including the application of the RNA Spike-In kit (Exiqon A/S, Denmark) to assess whether hemolysis or red blood cell contamination had occurred, as well as the efficiency of RNA isolation. The yield of miRNA obtained from the plasma samples was assessed by the inclusion of carrier RNA from the bacteriophage MS2. MiRNA expression levels were retrieved from a miRNA data set that had been generated by RNA sequencing of >390 human tissue and primary cell preparations (32).

### microRNA Profiling

Four microliters of total RNA was reverse transcribed to complementary DNA (cDNA) using the Exiqon Universal cDNA Synthesis Kit II. After 50-fold dilution, cDNA was mixed in equal proportion with 2 × Exiqon Exilent SYBR Green master mix and ROX Reference Dye (4 μl/2 ml) (Thermo Fisher Scientific). Samples loaded onto Exiqon Human Serum/Plasma Focus microRNA PCR Panels, 384 well (V4.M) were ran on QuantStudio 12K Flex Real-Time PCR System (Applied Biosystems) using parameters specified in the Exiqon miRCURY LNA Universal RT microRNA PCR instruction manual. Raw PCR data were processed and analyzed using Exiqon GenEx qPCR analysis software (Version 6). Only microRNA assays with C_t_ ≤ 35 and expressed in at least 60% of the samples were counted. Samples with a high degree of hemolysis were identified using the ΔC_t_ between hsa-miR-23a-3p and hsa-miR-451a, and those with ΔC_t_ >7 were omitted from the analysis. The UniSp3 interplate calibrator was used to even out run-to-run variation among panels. A ΔC_t_ of 1 between assays and a no-template negative control was used as a cutoff to eliminate spurious amplifications. The global average of all expressed microRNAs with C_t_ <35 was used to normalize individual assays. Statistical analysis was performed using an unpaired, two-tailed *t* test with a confidence interval of 95% (*p* ≤ 0.05). Bonferroni correction was applied, and advanced multiple testing was not performed.

### Statistics

For this pilot study, no information about changes in miRNA expression levels was available to allow for sample size calculation. For pilot studies such as this one, Birkett and Day (33) have suggested a minimum of 20 degrees of freedom to allow an estimation of variance from which a larger trial could be powered. Therefore, in this study, 29 subjects in each group were recruited. Statistical analysis was performed using SPSS (v22, Chicago, IL). Normality of continuous data was assessed visually and statistically using the Shapiro–Wilk test. For continuous data, the descriptive data are here presented as mean ± standard deviation (SD); for categorical data, they are presented as *n* (%). As appropriate, *t* tests or Mann–Whitney tests were used to compare means/medians. Spearman's correlation test was used to assess associations. Statistical significance was set below α = 0.05. Before analysis, mean normalization was performed using the global mean for each miRNA. This was accomplished using GenEx software (provided by Agilent for use with the 2100 Bioanalyzer instrument); normalization achieved a global mean for all miRNAs with C_t_ <35. An unpaired *t* test was used in order to test any paired changes in miRNA expression levels between the PCOS and control women. False discovery rates (FDRs) of *q* < 0.05 (34) were taken as significant. Ingenuity Pathway Analysis (Qiagen) was undertaken; pathways overrepresented by the FDR-significant miRNA changes at *q* < 0.05 (34) were identified.

## Results

Baseline data for the 58 patients, 29 PCOS and 29 normal controls, are shown in Table 1, where it can be seen that patients were overweight, age and BMI matched, and differed significantly in their insulin levels, insulin resistance (HOMA), testosterone, free FAI, and AMH.

**Figure 1:**
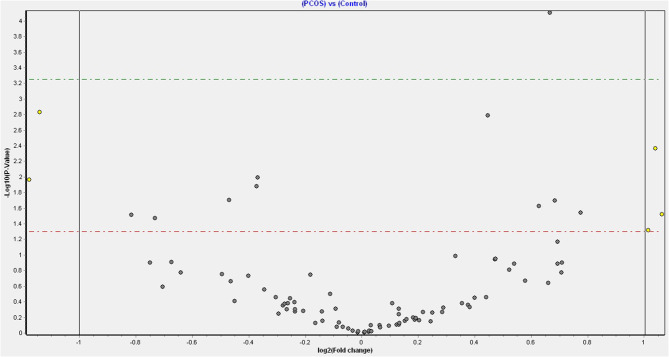
Volcano plot of fold changes in miR and significance level.

One hundred seventy-six miRNA were detected, of which 15 differed significantly (Table 2, Figure 1) between normal women and PCOS. The top 9 of these (*p* < 0.02) were miR-486-5p, miR-24-3p, miR-19b-3p, miR-22-3p, miR-19a-3p, miR-339-5p, miR-185-5p, miR-101-3p, and miR-let-7i-5p. There was no association of the top 9 miRNA that differed with BMI, androgen levels, insulin resistance, or AMH in either PCOS or normal women (Supplementary Table 1).

**Table 2:** Significant microRNA (*n* = 15) in patients in the follicular phase of menstrual cycle for women with (*n* = 29) and without (*n* = 29) polycystic ovarian syndrome (PCOS); patients with PCOS had been amenorrheic for a minimum of 6 weeks.

**(PCOS) vs. (control)**	**Fold change**	***p* value**
hsa-miR-486-5p	1.58665	0.00007868
hsa-miR-24-3p	−2.20464	0.001472633
hsa-miR-19b-3p	1.36323	0.00162241
hsa-miR-22-3p	2.05511	0.00434351
hsa-miR-19a-3p	−1.29124	0.010221734
hsa-miR-339-5p	−2.26205	0.010837199
hsa-miR-185-5p	−1.29397	0.013286524
hsa-miR-101-3p	−1.38422	0.019903021
hsa-let-7i-5p	1.60721	0.020206774
hsa-miR-21-5p	1.5459	0.023646336
hsa-miR-424-5p	1.71173	0.028857643
hsa-miR-151a-3p	2.08966	0.030324572
hsa-miR-148b-3p	−1.76088	0.030526907
hsa-miR-191-5p	−1.66057	0.034023782
hsa-miR-199a-3p	2.02076	0.048438053

**Figure 2:**
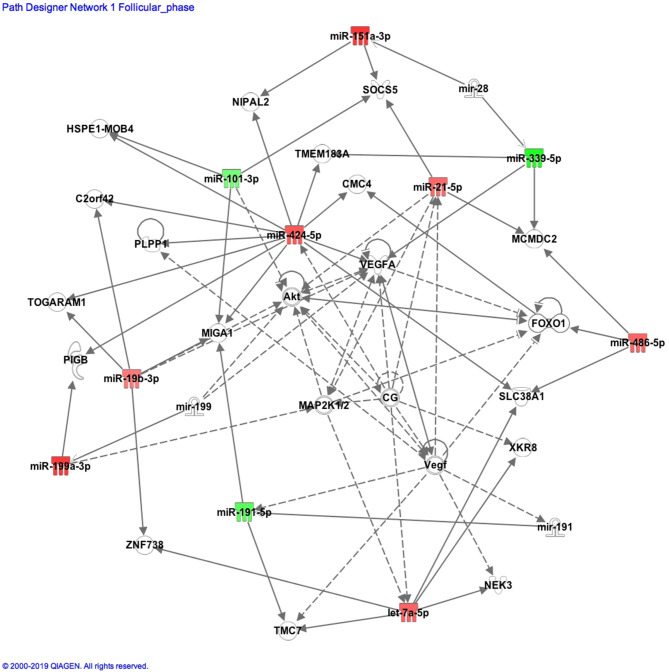
Pathway connections for the organismal damage and reproductive pathways and significantly changed microRNAs focused around the vascular endothelial growth factor A (VEGFA) gene. The miR in green showed a significant increase, while the miR in red showed a significant decrease when comparing those women with (*n* = 29) and without (*n* = 29) polycystic ovarian syndrome (PCOS).

A more recent study has shown that sets of microRNAs can coordinately act in distinct biological pathways, thereby revealing an emerging concept of regulatory microRNA networks (35). When Ingenuity Pathway Analysis was undertaken to look at the functional relationships, the significant miRs mapped to the pathways of reproductive abnormalities as shown in Figure 2. Notably, all nine microRNAs revealed functional connectivity as part of a regulatory network of genes predominantly associated with central regulators of insulin signaling and glucose metabolism such as the AKT-FOXO1 circuit (36). In addition, the generated pathway also suggested vascular endothelial growth factor (VEGF) to be associated with the altered microRNA functions, which is important because VEGF contributes to the pathogenesis of PCOS (37).

## Discussion

Altered microRNAs in circulation reflect significant changes in peripheral tissues and are considered as potential biomarkers similar to enzymes and cytokines in obesity-associated metabolic diseases (38). The key results of this study were the significant alterations in miRNAs in anovulatory PCOS women compared to their age- and BMI-matched control counterparts, all of whom were documented to be in the follicular phase of their menstrual cycle. In the follicular phase, in PCOS patients compared to controls, there were 15 miRNAs that differed significantly; the 9 most significant were miR-486-5p, miR-24-3p, miR-19b-3p, miR-22-3p, miR-19a-3p, miR-339-5p, miR-185-5p, miR-101-3p, and miR-let-7i-5p.

Given that the subjects were BMI matched, then it is perhaps unsurprising to see that the miRNAs that have been reported to be related to BMI, such as miR-21, miR-27b, miR-103, and miR-155 (23), did not differ in this study between groups. miR-93 has been implicated in downregulating the expression of the insulin-sensitive glucose transporter GLUT4 in adipose tissue (29) and has been shown to be overexpressed in adipose tissue (29). In addition, the discrepancy with the literature may be in part due to the differing unmatched populations that have been studied and the potential confounding influence of miRNA changes through the menstrual cycle that are still undefined.

While some of the miRNAs have been associated with insulin resistance such as miR-93, much of the validation of these miRNAs described have been done in the cancer-related field. Previous studies have shown that miR-486-5p plays a role as a tumor suppressor of nonsmall cell lung cancer (NSCLC) (1), hepatocellular carcinoma cells (2), and leukemia cells, where it induces apoptosis by targeting FOXO1 (3); it also prevents the migration, invasion, and endothelial-to-mesenchymal transition (EMT) by regulating Smad2 in breast cancer (4). MiR-24-3p was reported to suppress cellar proliferation in hepatocellular carcinoma (5) as well as promote cellular invasion and migration in nasopharyngeal carcinoma by targeting TEL2 (6) and in lung cancer by targeting SOX7 (7). Studies on hepatocellular carcinoma have shown that miR-19a-3p contributes to tumor metastasis and regulates cell growth via the PTEN/Akt and PI3KIP1/Akt pathways, respectively (8, 9). MiR-19a-3p downregulation in prostate cancer activates the transforming growth factor beta (TGF-β) signaling pathway, which stimulates migration, invasion, and bone metastasis (10). Reports on miR-19b-3p demonstrated that it inhibits cell proliferation in breast cancer via the PI3K/Akt pathway (11), whereas, by contrast, it promotes cell proliferation in colon cancer via targeting SMAD4 (12). A study in human cervical squamous carcinoma reported that miR-22-3p causes inhibition of cell apoptosis by targeting the eIF4EBP3 gene (13) and suppresses cell proliferation in hepatocellular carcinoma and arterial smooth muscle by regulating SP1 and HMGB1, respectively (14, 15). Studies investigating the role of miR-339-5p indicate that it inhibits tumor invasion in breast cancer (16) and in hepatocellular carcinoma by targeting ZNF689 (17, 18); by regulating EMT, it inhibits metastasis of NSCLC (19). However, it was reported to promote stem cell leukemia syndrome development through the downregulation of the BCL2L11 and BAX proapoptotic genes (20). MiR-185 was reported to induce potent autophagy by AKT signaling in hepatocellular carcinoma (21), whereas in human prostate cancer, it was reported to promote apoptosis (22). Reports indicate that miR-101-3p targets Pim-1 in salivary gland adenoid cystic carcinoma to enhance the sensitivity to chemotherapy and suppress cell proliferation and invasion (23); miR-101-3p was also reported to target MALAT-1 in nonsmall cell lung cancer to inhibit growth and metastasis (24). Recent studies have found that the role of let-7i-5p is to suppress cellular migration and proliferation via HMGA1 gene targeting in bladder cancer (25) and kallikrein-related peptidase 6 in colon cancer (26). Moreover, the inhibition of let-7i was reported to enhance progesterone-induced functional recovery in an ischemic mouse model (27).

Differing types of microRNA can coordinate in biological pathways, indicating their regulation of microRNA networks (35). The Ingenuity Pathway Analysis revealed that all of the top miRNAs reported had functional connectivity as part of a regulatory network of genes predominantly associated with central regulators of insulin signaling and glucose metabolism such as the AKT-FOXO1 circuit (36). Furthermore, the microRNAs reported here were related to the pathway associated with the vascular endothelial growth factor A (VEGFA) gene, which is associated with genetic alterations of vascular endothelial growth factor (VEGF), and suggests that VEGF has a role in the pathogenesis of PCOS as a candidate locus (37). Genetic variations in the VEGFA gene contribute to VEGF secretion and PCOS.

For VEGFA, the single nucleotide polymorphism (SNP) rs3025020 was significantly higher in PCOS cases than in control women, while increased fasting insulin and HOMA-IR and bioactive testosterone were linked with rs3025020 (37). This has been recently repeated in a Chinese population (39). A systematic review suggested that VEGF was involved in the pathophysiology of PCOS and, in particular, the development of ovarian hyperstimulation syndrome (40). Our data indicate that both miR-339-5p and miR-424-5p can directly influence VEGF expression and potentially contribute to abnormal VEGF level and function in PCOS. Further studies, particularly the overexpression of the mRNA in animal models of PCOS, would yield greater insights into the function of these miRNAs and their role in the pathogenesis of PCOS.

Quantitative polymerase chain reaction (qPCR) is the preferred method for determining miRNA expression. It is considered to be more accurate, simpler to perform, and delivers superior reproducibility when compared to other methods (hybridization or sequencing-based technologies) (41). It should be noted that most reported changes in circulating microRNAs in metabolic disorders fall within the same range of 1.5- to a maximum of 4-fold changes (42) as found here, unless a much more severe pathophysiological condition is present, resulting in significant tissue damage and cell death (43). Thereby, owing to their high abundance and stability, a twofold difference in body fluid microRNAs is considered as significant (38). The microRNA levels in this study were analyzed using the most reliable PCR-based platform developed by Exiqon, which relatively performs at least as well as in RNA sequencing (44). A deeper analysis of the reflected changes in identified microRNAs would provide a better understanding of the cellular mechanisms associated with PCOS, and this would require intense functional cell-based studies.

The strengths of this study were that the subjects were age and sex matched and that the PCOS population was homogeneous, demonstrating all three diagnostic features. It has been reported that PCOS subjects with all three diagnostic features have a more metabolic phenotype independent of obesity (45); therefore, with this exaggerated phenotype, if a relationship existed between the metabolic parameters and miRNA, it would be apparent. As a pilot study, one limitation was the relatively small number of subjects available. Despite this, the results clearly showed that miRNA appeared to differ between control women in the follicular phase of the menstrual cycle and anovulatory PCOS women.

In conclusion, these data indicate that both miR-339-5p and miR-424-5p can directly influence VEGF expression and potentially contribute to abnormal VEGF level and function in PCOS; miRNAs were found to differ between women without PCOS in the follicular phase of the menstrual cycle and anovulatory PCOS women, but when age and BMI were matched, there were no associations with AMH and the metabolic parameters.

## Data Availability Statement

The data that support the findings of this study are available from the corresponding author upon request.

## Author Contributions

AB contributed to data analysis and wrote the manuscript. SHN-S performed the miR measurements. VR, TS, RD, NG, MB, SD, and SH contributed to data analysis. TS supervised sample collection. SA designed the studies, supervised the work, contributed to data analysis, and was involved in preparation of the manuscript. SA is the guarantor of this work and, as such, had full access to all the data in the study and takes responsibility for the integrity of the data and the accuracy of the data analysis.

## Conflict of Interest

RD is currently employed by the company AstraZeneca. The remaining authors declare that the research was conducted in the absence of any commercial or financial relationships that could be construed as a potential conflict of interest.
