# Corrigendum: microRNA Expression in Women With and Without Polycystic Ovarian Syndrome Matched for Body Mass Index

**DOI:** 10.3389/fendo.2020.00515

**Published:** 2020-07-30

**Authors:** Alexandra E. Butler, Vimal Ramachandran, Thozhukat Sathyapalan, Rhiannon David, Nigel J. Gooderham, Manasi Benurwar, Soha R. Dargham, Shahina Hayat, S. Hani Najafi-Shoushtari, Stephen L. Atkin

**Affiliations:** ^1^Diabetes Research Center (DRC), Qatar Biomedical Research Institute (QBRI), Hamad Bin Khalifa University (HBKU), Qatar Foundation (QF), Doha, Qatar; ^2^Division of Research, Weill Cornell Medical College-Qatar, Qatar Foundation, Education City, Doha, Qatar; ^3^Academic Diabetes, Endocrinology and Metabolism, Hull York Medical School, University of Hull, Hull, United Kingdom; ^4^Department of Surgery & Cancer, Faculty of Medicine, Imperial College, London, United Kingdom; ^5^Department of Cell and Developmental Biology, Weill Cornell Medicine, New York, NY, United States; ^6^Royal College of Surgeons Ireland, Busaiteen, Bahrain

**Keywords:** microRNA, polycystic ovarian syndrome, follicular phase, menstrual cycle, insulin resistance, androgens

An author name was incorrectly spelled as **Sathypalan**. The correct spelling is **Sathyapalan**.

The authors apologize for this error and state that this does not change the scientific conclusions of the article in any way. The original article has been updated.

