# Supplementary material for: microRNA Expression in Women With and Without Polycystic Ovarian Syndrome Matched for Body Mass Index
Source: Front Endocrinol (Lausanne). 2020 Apr 28;11:206. doi: 10.3389/fendo.2020.00206 (PMC7199502; doi:10.3389/fendo.2020.00206)

**Supplementary table 1.** Relationship between the top 9 miRNAs that differed significantly between PCOS and control women with key demographic and biochemical parameters. There was no association of the top 9 miRNA that differed with BMI, androgen levels, insulin resistance or AMH in either PCOS or normal women.


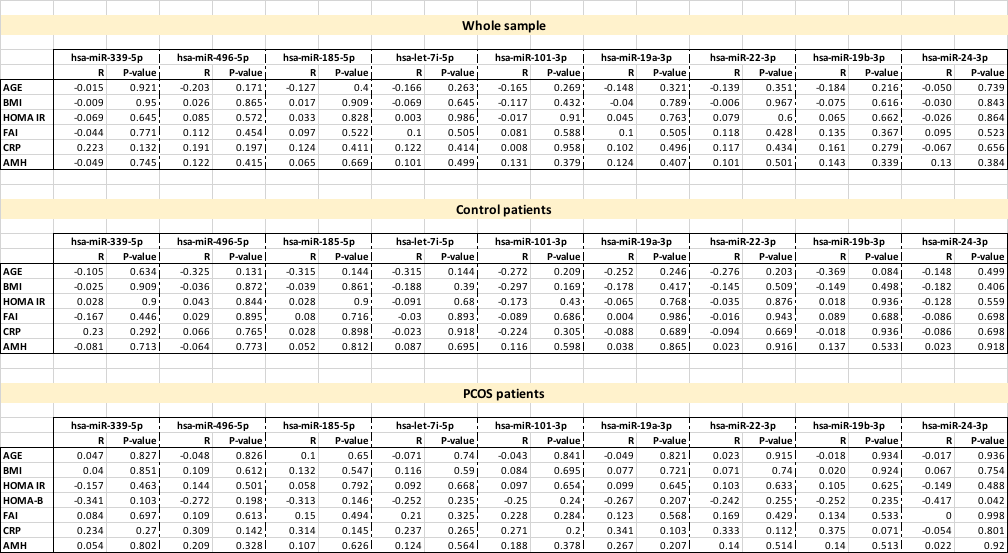

Supplement: Supplementary file 1 [file Table_1.docx]
